# MR spectroscopy in breast cancer metabolomics

**DOI:** 10.1002/ansa.202000160

**Published:** 2021-05-03

**Authors:** Uma Sharma, Naranamangalam R. Jagannathan

**Affiliations:** ^1^ Department of NMR & MRI Facility All India Institute of Medical Sciences New Delhi India; ^2^ Department of Radiology, Chettinad Hospital & Research Institute Chettinad Academy of Research & Education Kelambakkam India; ^3^ Department of Radiology Sri Ramachandra Institute of Higher Education and Research Chennai India; ^4^ Department of Electrical Engineering Indian Institute of Technology Madras Chennai India

**Keywords:** biomarker, breast cancer, ex vivo, in vitro, in vivo, in vivo magnetic resonance spectroscopy (MRS), metabolomics, therapeutic response

## Abstract

Breast cancer poses a significant health care challenge worldwide requiring early detection and effective treatment strategies for better patient outcome. A deeper understanding of the breast cancer biology and metabolism may help developing better diagnostic and therapeutic approaches. Metabolomic studies give a comprehensive analysis of small molecule metabolites present in human tissues in vivo. The changes in the level of these metabolites provide information on the complex mechanism of the development of the disease and its progression. Metabolomic approach using analytical techniques such as magnetic resonance spectroscopy (MRS) has evolved as an important tool for identifying clinically relevant metabolic biomarkers. The metabolic characterization of breast lesions using in‐vivo MRS has shown that malignant breast tissues contain elevated levels of choline containing compounds (tCho), suggesting rapid proliferation of cancer cells and alterations in membrane metabolism. Also, tCho has been identified as one of the important biomarkers that help to enhance the diagnostic accuracy of dynamic contrast enhanced magnetic resonance imaging and also for monitoring treatment response. Further, metabolome of malignant tissues can be studied using ex vivo and in vitro MRS at high magnetic fields. This provided the advantage of detection of a large number of compounds that facilitated more comprehensive insight into the altered metabolic pathways associated with the cancer development and progression and also in identification of several metabolites as potential biomarkers. This article briefly reviews the role of MRS based metabolic profiling in the discovery of biomarkers and understanding of the altered metabolism in breast cancer.

## 1 INTRODUCTION

Breast cancer is a major healthcare concern for women globally.^1^ Early diagnosis of breast cancer plays an important role in effective therapeutic management and in improving survival and quality‐of‐life. The radiological imaging techniques such as mammography and ultrasound have been used for screening of breast lesions. However, mammography has the limitation of detecting lesions in dense breast, while identification of micro‐calcifications and overlap in morphology of benign and malignant lesions limits the use of ultrasound.^2^, ^3^ Dynamic contrast enhanced magnetic resonance imaging (DCEMRI) has become a valuable imaging technique for preoperative staging, therapeutic monitoring and detection of breast cancer recurrence.^3^ Recent breast screening studies have reported sensitivity of DCEMRI to be between 75.2% and 100% and specificity in the range of 83‐98.4% in women having hereditary and familial risk of breast cancer.^4^, ^5^

Over the years metabolomics has emerged as an innovative powerful tool in the discovery of disease biomarkers and for providing an understanding of the disease biology.^6^, ^7^, ^8^, ^9^ It is known that the malignant transformation of a cell is a resultant of several complex events like alterations in the several regulatory pathways at the molecular level that is manifested as changes in the metabolism of a living system.^10^ Metabolomics approach is based on the comprehensive measurement of changes in all small molecular weight metabolites in a living system.^6^, ^7^ The concentrations of metabolites are sensitive to changes in the metabolic pathways therefore, metabolome of an individual represents their phenotype in healthy and diseased states. The alterations in metabolite levels has the potential to provide deeper understanding of the phenotypic changes resulting from genetic alterations, pathological, physiological, environmental, and toxicological influences.^6^, ^7^

Magnetic resonance spectroscopy (MRS) and mass spectrometry (MS) have been the two major techniques to study cancer metabolomics.^8^, ^9^ Each of these analytical methods has their unique advantages and disadvantages. The MRS has several advantages; it provides rapid analysis by simultaneous detection of different classes of compounds without the need for separation and derivatization of compounds.^6^, ^7^, ^8^, ^9^ Further, MRS is nondestructive and has high reproducibility compared to MS analysis. These features have made MRS an important tool for studying various applications of metabolomics.^6^, ^7^, ^8^, ^9^ However, unlike MRS, MS based analysis requires separation of individual compounds using techniques like liquid chromatography and gas chromatography. Following chromatographic separation, compounds are ionized and then analyzed based on their charge/mass ratio through MS analyzer. In recent years, several studies have used LC‐MS and GC‐MS as a promising tool for characterization of metabolites in breast cancer.^8^, ^9^, ^11^, ^12^, ^13^ The disadvantage of MRS is its poor sensitivity compared to MS, however, the use of high field magnets (at proton (^1^H) resonance frequency greater than 1.0 GHz) and cryo probes have markedly enhanced the sensitivity of MR spectroscopic analysis.^14^, ^15^ It is possible to detect metabolites in picomole concentration with special probes.^14^, ^15^ A comparison of MS and MRS techniques is presented in the Table 1.

**TABLE 1:** Comparison of magnetic resonance spectroscopy (MRS) and mass spectrometry (MS)

Characteristics	MRS	MS
Biological Specimen/Sample	MRS has three variants: In‐vivo MRS: performed on living humans, animals Ex‐vivo MRS: performed on excised intact tissue In‐vitro MRS: performed on tissue extracts, biological fluids like blood plasma/sera, urine, saliva, cell lines.	Performed on tissue/ cell line extracts and biofluids like blood sera/plasma and urine.
Nature	Non‐destructive, sample can be used for repeated analysis. In‐vivo MRS is appropriate for longitudinal studies.	Destructive in nature, sample cannot be recovered and used for other analysis.
Sample preparation	Minimal sample preparation required	Complex sample preparation requires chromatographic separation and derivatization of compounds.
Acquisition of data	The data acquisition is fast using 1D ^1^H‐MR spectroscopy. Non‐selective, all ^1^H containing metabolites are detected in one 1D experiment.	Not as fast as MRS, as different ionization techniques and detectors are required.
Sensitivity	Low sensitivity, however, with the use higher magnetic field, microprobes, cryoprobes and nanoprobes, the sensitivity is improved, and compounds in picomoles concentration can be detected.	Highly sensitive and compounds in the range of femtomolar concentration can be detected.
Reproducibility	High reproducibility	Compared to MRS, less reproducibility
Quantitation	Quantitation is easier as the intensity of peak is directly proportional to the concentration of metabolite and number of nuclei contributing to the peak.	The intensity of the MS peak has dependence on ionization efficiency and is not correlated with the concentration of metabolite.
Targeted/Untargeted	Can be used for both types of analysis	Better for targeted analysis

MRS based metabolomics has been widely used to identify biomarkers and to gain an insight into the altered biochemistry associated with the breast cancer development and progression.^16^, ^17^, ^18^, ^19^ In‐vivo MRS has evolved as a non‐invasive modality for evaluating the metabolic profile from a specific region of interest in a living system, facilitating understanding of the disease biochemistry and the discovery of biomarkers.^20^, ^21^, ^22^, ^23^, ^24^, ^25^, ^26^, ^27^, ^28^, ^29^, ^30^ Several in vivo MRS studies have shown that malignant tumors can be differentiated from benign lesions based on higher levels of choline containing compounds (tCho), which can serve as a biomarker of malignancy.^20^, ^21^, ^22^, ^23^, ^24^, ^25^, ^26^, ^27^, ^28^, ^29^, ^30^ Few studies have also shown the alterations in water‐to‐fat ratio (W‐F) and lipid composition in the breast cancer.^25^, ^31^, ^32^, ^33^, ^34^, ^35^, ^36^ However, overlap in the values of W‐F ratio between cancer and benign diseases limit its diagnostic utility.^25^ The changes in W‐F value have been found useful in monitoring the therapeutic response in breast cancer patients.^31^ The improved specificity of DCEMRI has been observed in combination with MRS of breast cancer.^26^, ^27^, ^37^, ^38^, ^39^

The ex‐vivo high‐resolution magic angle spinning (HRMAS) MRS has also been widely used for examining the metabolic profile of intact breast cancer tissue for assessing diagnostic biomarkers and understanding association of metabolite levels with prognosis.^40^, ^41^ Further MRS analysis of tissue extracts (biopsy/surgical),^42^, ^43^, ^44^ fine needle biopsy (FNAB)^45^ fine needle aspiration cytology (FNAC),^43^, ^46^, ^47^ nipple aspirate,^18^ axillary nodes,^48^, ^49^ and blood sera/plasma^17^, ^50^, ^51^ samples, using in‐vitro MR spectroscopy techniques have shown the potential of detecting large number of metabolites compared to in vivo, which facilitated identification of more number of potential biomarkers, which may have diagnostic and prognostic value. This review briefly describes the utility of in vivo, ex vivo, and in vitro MRS based metabolomics approaches in diagnosis and therapy of breast cancer with representative examples of the research work carried out from our laboratory and elsewhere in India with relevant international literature. This article has not been intended to review all the breast MRS literature, since the focus has been primarily on contributions from India. However, the interested readers may refer to other reviews for comprehensive details of MRS and MS based metabolomics approaches in breast cancer research.^8^, ^9^, ^27^, ^28^, ^37^, ^38^, ^39^, ^40^, ^41^, ^52^, ^53^, ^54^, ^55^

## 2 IN VIVO MR SPECTROSCOPY (MRS)

In vivo MRS provides information on the metabolites present in a defined volume of interest (VOI) in any organ such as breast. Most breast MRS studies have been performed using ^1^H nuclei due to its high sensitivity and natural abundance in living tissues. The in vivo MRS, is technically referred as image guided localized spectroscopy and it has two variants: namely, (a) single‐voxel spectroscopy (SVS), when the spectrum is acquired from a single voxel; and (b) MR spectroscopic imaging (MRSI) or also known as chemical shift imaging, using which spectra from multiple voxels are acquired simultaneously. The placement of voxel is guided using MR images acquired in three orthogonal planes, earlier. The SVS is less sensitive to patient motion and a spectrum of better quality is obtained which is suited for quantitative analysis than MRSI. However, MRSI provides the advantage of simultaneous assessment of the lesion and the normal parenchyma of breast as well as information on the spatial variation of metabolites within a tumor. Additionally, color coded metabolic maps can be generated for visual assessment of metabolite levels which could be developed as more useful tool in clinical settings. The pulse sequences which are commonly applied for localization in both single and multivoxel modes are the stimulated echo acquisition mode (STEAM) and point resolved spectroscopy (PRESS).^27^, ^28^, ^37^, ^38^, ^39^, ^52^, ^53^, ^54^, ^55^ Most in vivo MRS studies were carried out using a 1.5 T MR scanner, however, recently several studies have been reported using 3T MR scanners and few at 4T and 7T to gain the advantage of the increased sensitivity and spectral resolution.^56^, ^57^, ^58^, ^59^, ^60^, ^61^ Dedicated breast coils either single or bilateral are used for acquisition of breast MRS. For MR spectral acquisition, the patient is positioned in a prone position in the gantry of the magnet while the breast is fitted in the cup of the breast coil with additional cushions inside to reduce motion artifacts. The MR spectrum of breast acquired without the suppression of water and the lipid resonances provides information on the various lipid resonances and the water content in tumors. While the signal from choline containing metabolites (tCho) is observed when signals from both the water and the lipid resonances are suppressed. The various parameters that are calculated from the MR spectrum for characterizing breast malignancy are: W‐F ratio, water fraction, fat fraction,^25^, ^31^, ^32^, ^33^, ^34^, ^35^, ^36^ and tCho peak.^20^, ^21^, ^22^, ^23^, ^24^, ^25^, ^26^, ^27^, ^28^, ^29^, ^30^ The MRS of breast is performed at long echo times (TE above 100 ms). Though short echo time MRS provides high signal intensity of tCho peak, but at long echo times provides an advantage of better suppression of the huge lipid signal enabling better detection of tCho.^22^, ^23^, ^24^, ^25^, ^26^, ^37^

### 2.1 Lipid and water composition in the normal and malignant breast tissue

**FIGURE 1:**
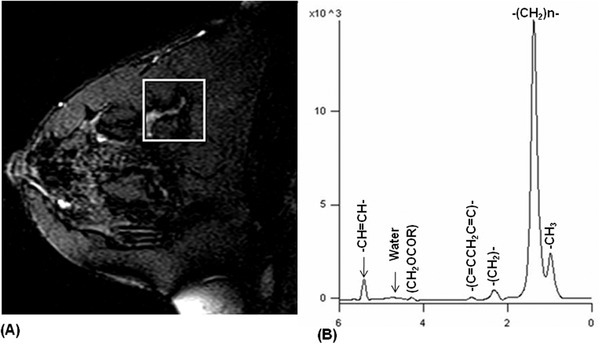
(**A**) T1‐weighted MR image of the normal breast from a volunteer showing the voxel position from which a single‐voxel ^1^H MR in vivo spectrum (**B**) was obtained without water and fat suppression (Reprinted with permission from John Wiley & Sons, Inc. from references # 37 and 38)

**FIGURE 2:**
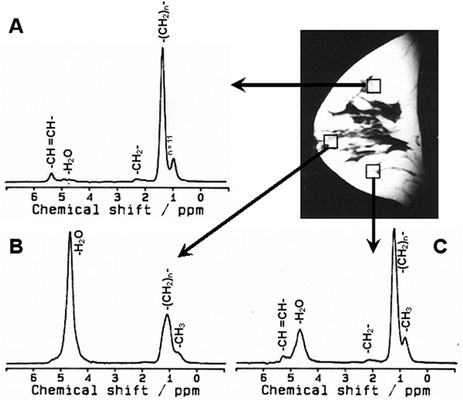
In vivo proton MR spectra acquired at TE = 135 ms from three different voxel (8 ml) locations within the normal breast of a 31 year old normal female volunteer. (**A**) Upper quadrant, (**B**) Para‐areolar region, (**C**) Lower quadrant (Reprinted with permission from Elsevier from reference # 62)

Normal breast is a glandular organ containing high amount of lipid and low water content. Figure 1A shows the voxel position in the T1 weighted image, while Figure 1B shows the typical ^1^H MR spectrum of the normal breast tissue of a healthy volunteer acquired without suppression of water and fat.^37^, ^38^ The spectrum shows an intense peak at 1.3 ppm pertaining to [–(CH_2_)_n_–] protons of the lipids and the peak at 0.9 ppm is due to the CH_3_ protons of glycerides. α‐methylene protons of the glyceride chain are assigned at 2.2 ppm while diallylic CH_2_ protons resonate at 2.7 ppm. The peak observed at 5.2 ppm is due to CH of glycerol backbone and olefinic protons. The water resonance is observed at 4.7 ppm. The ratio of the integral values of the major lipid peaks (at 1.3 ppm and 0.9 ppm) and the water peak provides the measure of the W‐F ratio. Our group investigated the changes in W‐F ratio of the normal breast parenchyma of healthy female volunteers during the five phases of the menstrual cycle in three anatomical regions of the breast, namely; upper and lower quadrants and para‐areolar region (see Figure 2).^62^ The W‐F ratio was found to be higher in para‐areolar region compared to upper and lower quadrants at all the phases of the menstrual cycle.^62^ Cyclic changes were seen in the W‐F ratio in the para‐areolar region during the menstrual cycle.^62^ The W‐F value was high (0.90 ± 0.41) in the proliferative phase, which showed a reduction (0.46 ± 0.21) in follicular and luteal phases (0.45 ± 0.25), while it increased during secretory (0.76 ± 0.61) and menstrual phases (0.87 ± 0.37).^62^ These findings suggested the heterogeneity of lipid composition within the breast. Further it suggested that the physiological factors like menstrual cycle affect the water and lipid compositions of the normal breast tissue. This also suggested the use of W‐F ratio for assessment of breast pathology requires a careful consideration of the time of menstruation and the location of the lesion within the breast.^62^

**FIGURE 3:**
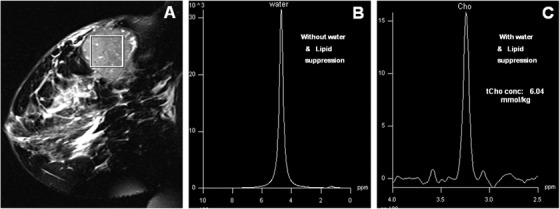
(**A**) MRI of a patient with locally advanced breast cancer showing the voxel position from which the single‐voxel ^1^H MR in‐vivo spectrum was obtained without water and fat (lipid) suppression (**B**) and with the suppression of water+fat (lipid) resonances (**C**) (Reprinted with permission from John Wiley & Sons, Inc. from reference # 38)

The T2‐weighted fat suppressed MR image of a breast cancer patient with histological type, infiltrating ductal carcinoma (IDC), is shown in Figure 3A, while the water and the fat unsuppressed spectrum from the VOI shown in the image is presented in Figure 3B. The MR spectrum from a malignant tumor showed a predominant water peak and a reduced lipid peak. Malignant breast tissues, thus found to have high W‐F value compared to the control and the normal unaffected breast tissues and from the contralateral breast tissues of patients.^25^, ^31^, ^32^ This indicated that the water content is considerably increased during malignancy.^25^, ^31^, ^32^, ^63^ Also, alterations in lipid content have been reported with the tumor progression.^64^ The W‐F parameter has also been used to monitor the response to neo‐adjuvant chemotherapy (NACT) in patients with locally advanced breast carcinoma (LABC). Data from our laboratory showed a reduction in W‐F ratio in patients receiving chemotherapy and the value was found to be associated with the reduction in the primary tumor size indicating that it can be used as a non‐invasive biomarker of tumor response.^31^, ^32^ However, the value of W‐F ratio showed significant overlap between benign and malignant tissues, suggesting its limited utility in the diagnosis of breast lesions.^65^ Recently, Wang et al suggested that composition of water and lipid and density in breast tissue is associated with the risk of breast cancer and it can be used for screening high‐risk women.^35^

Results of a recent study from our laboratory showed that malignant breast tissues are characterized with a lower fat fraction compared to normal breast tissues and benign lesions.^33^ Out of 68 breast cancer patients, 22 had ER+/PR+ hormone receptor status while 33 patients belonged to ER−/PR− status. Higher fat fraction was observed in patients with estrogen receptor negative (ER‐)/progesterone receptor (PR‐) status as compared to ER+/PR+ patients. Forty‐one patients showed Human epidermal growth factor 2 neu (HER2neu+), whereas 24 had HER2neu‐ status. The HER2neu+ tumors showed significantly higher fat fraction (median 0.14; range 0.02‐0.69) compared to HER2neu‐ (median 0.08; range 0.01‐0.25) breast tumors. A cut‐off value of 0.21 was calculated for fat fraction that provided 76% sensitivity and a 74.5% specificity in differentiating malignant and normal breast tissues while a cut off value of 0.18 was obtained for differentiation of malignant and benign lesions with a sensitivity of 75% and a specificity of 68.6%.^33^ In a recent study, using ^1^H MRS at 1.5 T, the levels of six lipid metabolites were determined and the differences in the composition of lipids were documented in benign and malignant lesions and among luminal A/B and other subtypes of breast cancer.^66^

### 2.2 Total choline (tCho) in normal, malignant, and benign breast tissues

**FIGURE 4:**
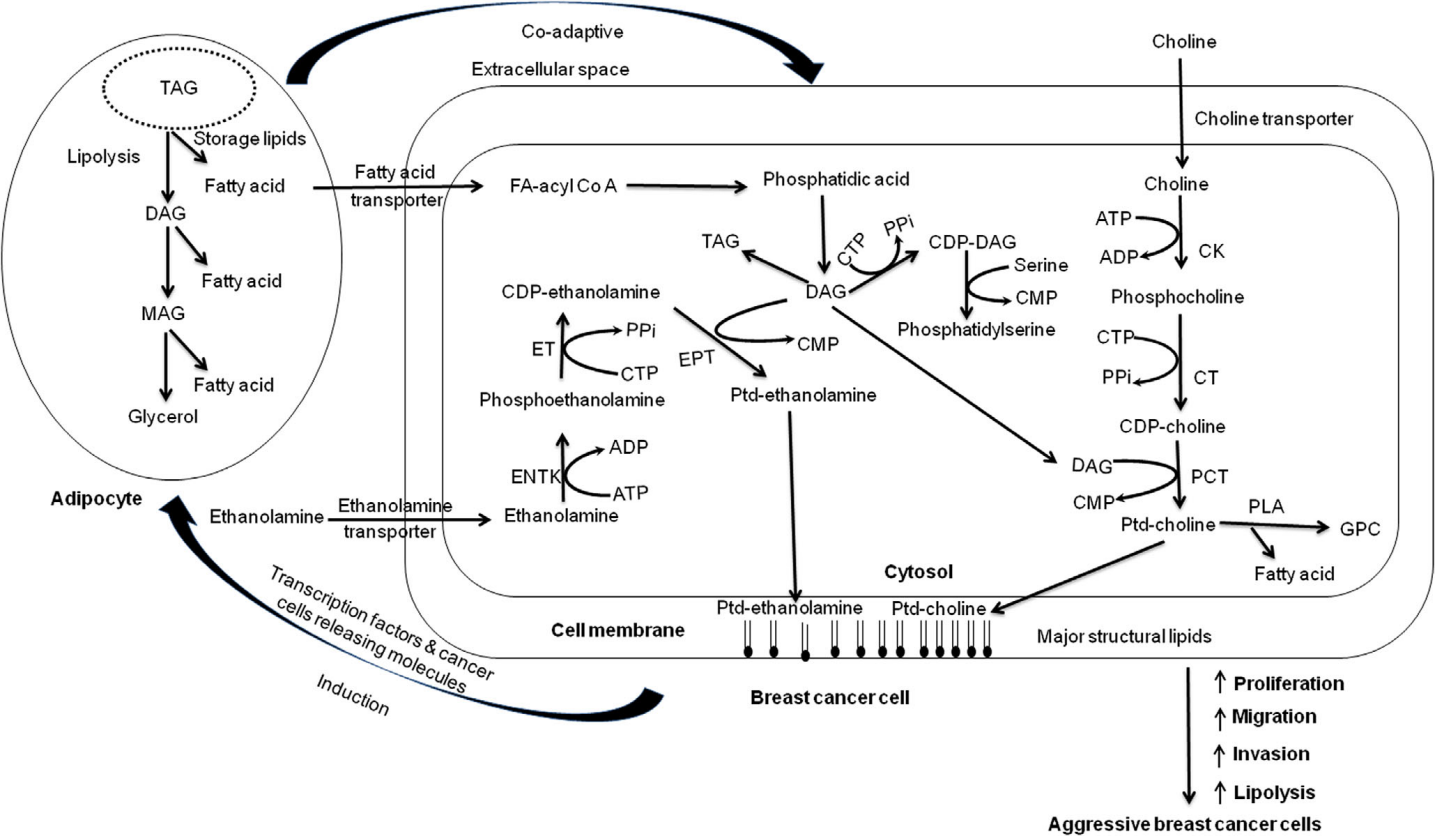
The role of metabolic reprogramming in breast cancer cells, their role in cancer and the induced co‐adaptive mechanism (Reprinted with permission from John Wiley & Sons, Inc. from reference # 27)

The ^1^H MR spectrum of a breast cancer patient suffering from IDC with water and lipid suppression shows a peak at 3.2 ppm due to choline‐containing compounds (tCho) (see Figure 3C)^38^. In our laboratory, we reported the absolute concentration of tCho in patients with early breast cancer (n = 31), LABC (n = 120), normal breast of healthy volunteers (n = 31), and benign breast lesions (n = 38), and also an association of tCho concentration with ER, PR, and HER2neu status. The concentration of tCho ranged from 0.76 to 21.2 mmol/kg in malignant breast tissues that was significantly higher compared to the benign lesions (0.04 to 2.70 mmol/kg) and normal breast tissues of healthy volunteers (0.1 to 1 mmol/kg).^30^ The concentration of tCho was found to be higher in patients with early breast cancer (5.4 ± 3.7 mmol/kg) compared to LABC patients (3.8 ± 2.0 mmol/kg).^30^ This may be attributed to the presence of necrosis in advanced stage tumors due to insufficient supply of nutrients. The tCho levels were higher in triple positive and non‐triple positive patients compared to triple negative patients. The cut‐off value for discriminating malignant and benign lesions was found to be 2.54 mmol/kg. Our results demonstrated that the quantitative assessment of tCho may be used in the clinical settings for diagnosis of breast lesions.^30^ A meta‐analysis of pooled MRS data from five initial studies that included the data from our laboratory as well, showed that the use of detection of tCho signal has a sensitivity of 83% and a specificity of 85% in differentiating breast cancer from benign lesions.^26^ The subgroup analysis in younger patients below the age of 40 years resulted in higher sensitivity of 100% and a specificity between 89% and 100% in this meta‐analysis.^21^, ^22^, ^23^ The tCho peak has been found to have contribution from several Cho containing compounds such as free choline (Cho), phosphocholine (PC), and glycerophosphocholine (GPC). However, it has been documented that increased signal of tCho is due to an increase in PC content in tumors.^29^, ^67^, ^68^, ^69^ Higher levels of PC has been found to be related to increased membrane synthesis required for tumor proliferation and suggested as hallmark of cancer.^67^, ^68^, ^69^ A closer look at the biosynthetic pathway of two important phospholipids, phosphotidylcholine (PtdCho) and phosphotidylethanolamine (PtdEtn) provides an understanding of higher tCho seen in malignant tumors (Figure 4). These phospholipids are important constituents of adipocytes. PC is formed from Cho after phosphorylation by creatine kinase (CK) enzyme and serves as a precursor for synthesis of PtdCho.^67^, ^68^, ^69^ It has been reported that stimulation of cells by fetal serum, growth factors, hormones, or tumor promoters can induce activation of CK that leads to enhanced phosphorylation of Cho to PC. This enhances the levels of PC that leads to increase in the biosynthesis of PtdCho.^70^ Further, it has been reported that cancer cells have faster transport of Cho in a study comparing transport of Cho in MCF‐7 breast cancer cell lines and normal mammary epithelial cells.^71^

Baltzer and Dietzel in 2013 performed a systematic review and meta‐analysis for estimating diagnostic performance of breast MRS in distinguishing between malignant and benign lesions. They included 19 breast MRS studies performed at 1.5 T and 3.0 T in a total of 1183 patients with 1198 lesions (malignant n = 773, benign n = 452). They reported a pooled sensitivity and a specificity of 73% and 88%, respectively noting no significant influences of higher magnetic field strength, post‐contrast acquisition and quantitative versus qualitative MRS evaluation.^54^ A meta‐analysis reported by Cen and Xu in 2014 included data of 18 SVS MRS studies of breast cancer patients and patients with benign diseases. This analysis included 750 malignant and 419 benign lesions.^55^ The pooled sensitivity obtained was 71% and the specificity was 85% for breast MRS. This analysis pointed toward standardization of the protocol for MRS acquisition among centers and also recommended a multicenter trial.^55^

### 2.3 tCho in lactating and normal breast tissues

**FIGURE 5:**
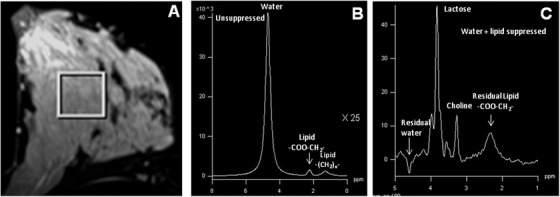
T2‐weighted fat suppressed axial image (**A**) from the normal breast tissue of a lactating women volunteer showing a voxel location of size 20×20×20 mm^3^ (**B**) corresponding ^1^H MR spectrum obtained without water and lipid suppression showing the water and lipid peaks. (**C**) ^1^H MR spectrum obtained with water and lipid suppression showing the residual water and lipid along with the tCho and the lactose peaks (Reprinted with permission from John Wiley & Sons, Inc. from reference # 72)

With the technological advances in magnets, breast coils and pulse sequences, a number of studies also reported the tCho signal in normal and lactating breast tissues.^21^, ^24^, ^29^ This raised a question on the use of tCho as a marker of breast malignancy. We performed a systematic breast MRS study on patients with LABC and normal healthy lactating women volunteers.^72^ The concentration of tCho was determined and spectral characteristics were compared between the two groups. The tCho peak was observed in all patients with LABC and the concentration was 3.51 ± 1.72 mmol/kg. In lactating women, tCho peak was detected in 10 out of 12 volunteers, with a concentration value of 3.52 ± 1.70 mmol/kg, which is similar to that observed in malignant lesions (Figure 5).^72^ Higher tCho levels in lactating breast tissues is due to the lactogenesis process. In neonates the choline obtained through mother's milk and is required for normal growth.^73^ However, a peak corresponding to lactose sugar was also observed in these volunteers, suggesting that this spectral characteristic is unique to lactating breast tissues.^72^ Further, it was documented through careful referencing by Stanwell et al that tCho peak observed in lactating breast is mainly due to GPC and not due to PC as observed in malignant lesions.^29^ Additionally, another MR parameter namely the apparent diffusion coefficient determined by diffusion MRI, could be used for differentiating lactating breast tissues from malignant tissues.^72^ The value of apparent diffusion coefficient was higher in lactating breast than in malignant breast lesions.

### 2.4 tCho and molecular markers

**FIGURE 6:**
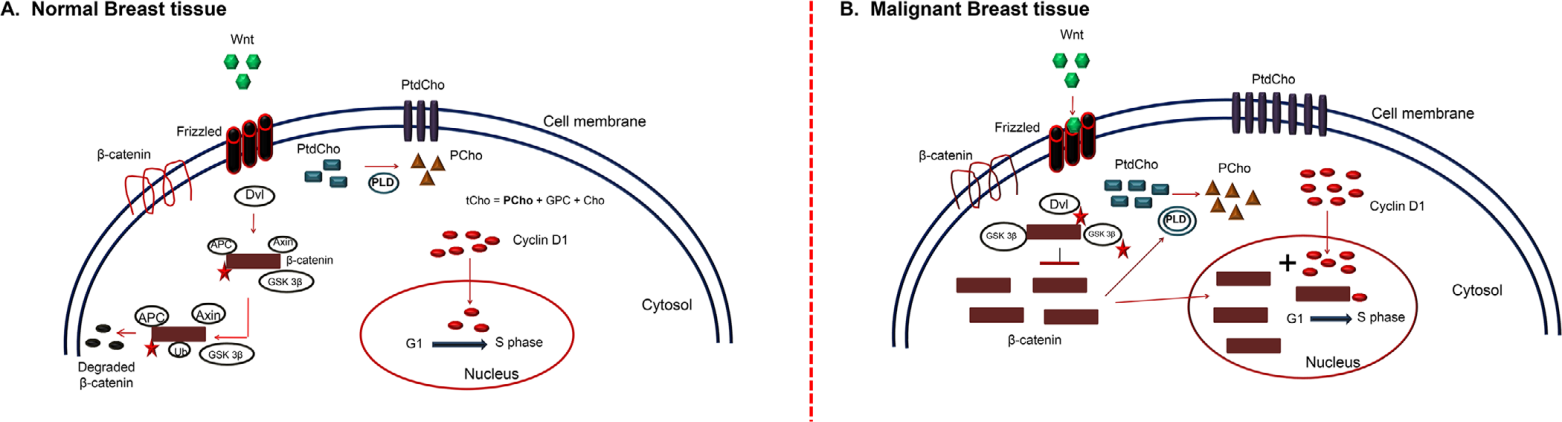
Schematic representation of the link between the choline synthesis and the Wnt‐mediated β‐catenin pathway. (**A**) Normal breast tissue: In a normal breast tissue Wnt (green polygon) signaling is absent and β‐catenin (brown rectangles) level is maintained low in the cell cytosol due to phosphorylation (red stars) and degradation of β‐catenin. Also, cyclin D1 (red circles) translocates to the nucleus at a reduced rate to participate in G1 to S phase transition. The cell division rate is regulated. Phosphatidyl choline (PtdCho; blue rectangle) which is a membrane phospholipid, converts into phosphocholine (PCho; brown triangle) in the cytosol by the activity of phospholipase D (PLD; grey circle). (**B**) Malignant breast tissue: During malignancy, Wnt signaling is active and hence β‐catenin increases in the cytosol that can translocate to the nucleus and bind to cyclin D1 (brown rectangle + red circle), to increase the rate of cellular transcription. With the increase in cellular proliferation, membrane requirement for PtdCho increases that in turn leads to increased PLD activity (double grey circle). PCho increases in the cell cytosol, thereby increasing the tCho levels. Increased cytosolic β‐catenin levels also increase the activity of PLD (Reprinted with permission from John Wiley & Sons, Inc. from reference # 74)

There has been an increasing interest in understanding the molecular mechanism of elevation of tCho seen in breast cancer patients. The molecular mechanism underlying tCho elevation in malignant tissues of breast cancer patients was recently reported by our group. An association of tCho with the expressions of β‐catenin and cyclin D1 proteins was documented (Figure 6).^74^ The Wnt/ β‐catenin pathway is a signal transduction pathway that regulates several cellular processes.^75^ It has been reported that activation of this pathway stimulate cell proliferation and associated with poor survival.^76^ In our study, the β‐catenin expressions was higher both in cytosolic and nuclear fractions in malignant tissues compared to benign and non‐involved tissues. In malignant tissues, a positive correlation was found between tCho and cytosolic and nuclear expressions of β‐catenin and cyclin D1. Patients with PR negative status showed higher cytosolic β‐catenin expression than positive patients. The findings may explain the molecular mechanism of elevated tCho and indication for β‐catenin pathway as a therapeutic target.^74^ The tCho level also showed correlation with nuclear grade and ER and PR status.^77^ A recent study showed a correlation between tCho levels and the expression of calcium‐sensing receptors.^78^

### 2.5 ^1^H MRS in evaluating therapeutic response

The potential of tCho as a non‐invasive biomarker has also been evaluated in the assessment of chemotherapeutic response of breast cancer patients. Reduction in tCho levels are reported in patients responding to chemotherapy or NACT suggesting the use of tCho as a non‐invasive marker of therapeutic response.^21^, ^24^, ^79^, ^80^, ^81^, ^82^ In an earlier study, we found that before therapy 10/14 cases showed tCho, while post‐therapy tCho signal was detected only in 7 patients indicating a positive response to NACT.^20^ We also evaluated the use of tCho signal‐to‐noise ratio (ChoSNR) in monitoring the tumor response. It was shown that in the patients who showed positive response to NACT, the level of ChoSNR and the tumor size reduced after three cycles of NACT compared to their pretherapy value. While in non‐responders, the values of the tumor size and the ChoSNR remained similar or increased compared to pre‐therapy value after NACT.^80^

**FIGURE 7:**
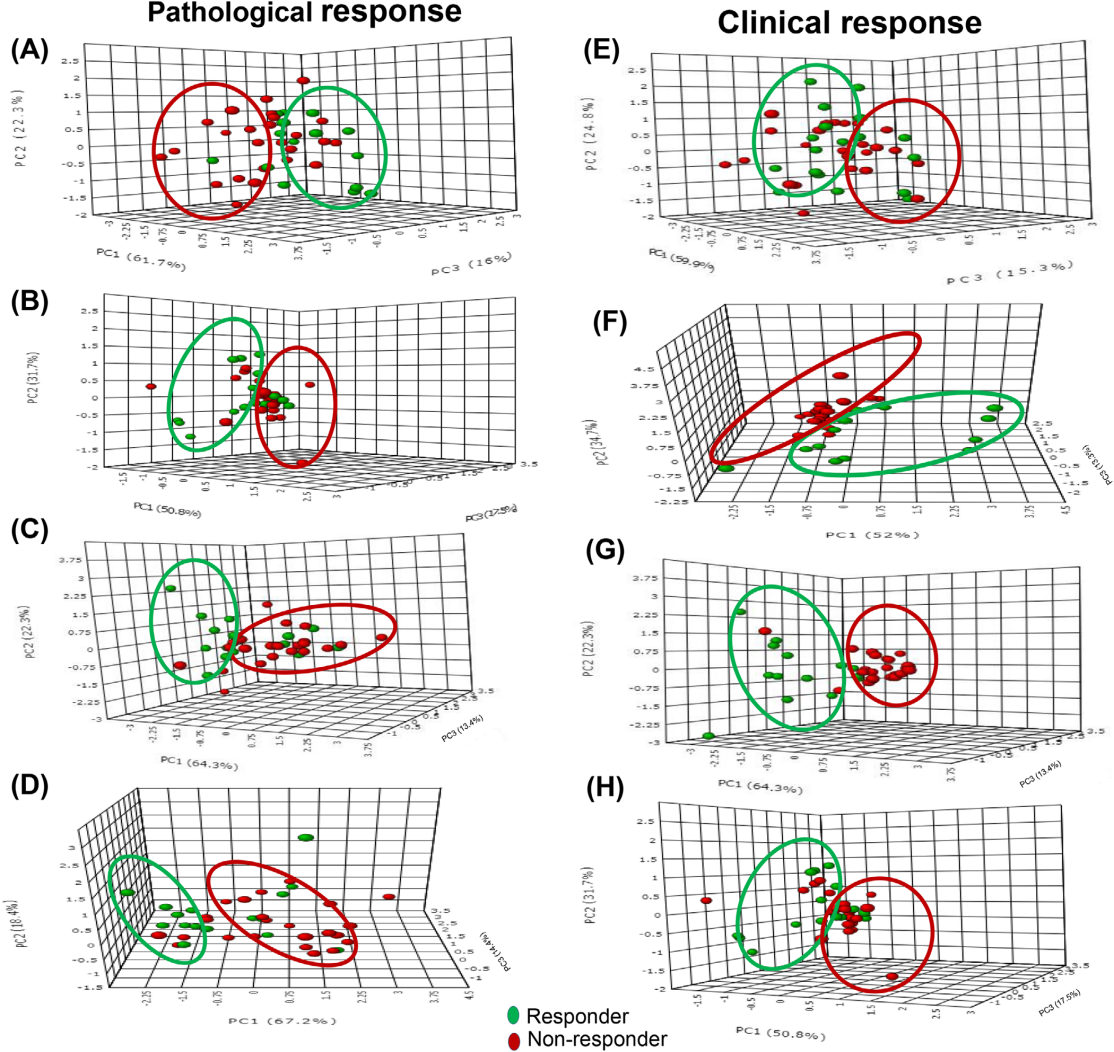
The 3‐D score plot (PC1‐PC3) of PCA analysis of multi‐parametric data (volume, ADC and tCho) in pathological responders and non‐responders at Tp0 (**A**) after Tp1 (**B**) Tp2 (**C**) and Tp3 (**D**) while (**E–H**) show the 3‐D score plot for clinical response (Figure as originally published in reference # 81: Uma Sharma, Khushbu Agarwal, Rani G. Sah, Rajinder Parshad, Vurthaluru Seenu, Sandeep Mathur, Siddhartha D. Gupta and Naranamangalam R. Jagannathan (2018). *Front. Oncol*. 15 August 2018 https://doi.org/10.3389/fonc.2018.00319)

Recently our group reported the role of multi‐parametric approach using three parameters namely, tCho, apparent diffusion coefficient determined from diffusion MRI and the tumor volume in monitoring both clinical and pathological responses of patients with LABC (n = 42) undergoing NACT (Figure 7).^81^ Our study showed that both tCho and ADC reduced as early as first cycle of NACT in both the clinical and the pathological responders while the tumor volume reduced only after second cycle of chemotherapy indicating that these two parameters can serve as an indicator of early response.^81^ Recently Drisis et al reported the value of tCho signal as early response predictor after NACT in 39 LABC patients.^83^ After first cycle of NACT, tCho level was found useful in predicting early response and pathological response. At the end of NACT, changes in tumor diameter, volume along with tCho levels predicted tumor response, while change in K^trans^ could predict only pathological response. The quantification of tCho was more sensitive for predicting pathological response in triple negative tumors.^83^ Bolan et al reported a multicenter analysis of ability of tCho as pathological response to NACT in the patients with LABC. MRS measurements were carried out before and after 20‐96 h of first cycle of NACT.^84^ The study enrolled 119 subjects, however, usable data could be obtained only for 29 cases. The decrease in tCho levels after 20‐96 h of first cycle of NACT showed poor ability to predict pathological response.^84^ Zhou et al recently evaluated the tumor response after the second and fourth cycles of NACT in breast cancer patients having nonconcentric shrinkage pattern.^85^ Significant change in tCho integral after fourth cycle was seen while there was no change in tumor size in responder and non‐responder groups. The sensitivity to detect response was 93.75%, with a positive predictive value of 78.9% and the AUC as 0.747 for tCho integral.^85^ Cho et al measured the changes in tCho by MRS and maximum and peak standardized uptake values and total lesion glycolysis by ^18^F‐fluorodeoxyglucose positron emission tomography and evaluated their ability to predict response to NACT in the 35 patients with LABC.^86^ Of the 35 patients, six had pathologic response while 29 were non‐responders. Mean percentage change in tCho, standardized uptake values and total lesion glycolysis of the patients with pathological response were larger than those of the non‐responders. The diagnostic accuracy for tCho was found to be comparable with other parameters.^86^

## 3 EX VIVO ^1^H MRS OF INTACT BREAST TISSUE

Ex vivo HRMAS proton MR spectroscopy has also been widely used to study metabolic processes in breast cancer tissue a non‐destructive manner. Most studies focused on assessing the diagnostic biomarkers, correlations of metabolite levels with prognosis and monitoring therapeutic response.^40^, ^41^ The advantage with HRMAS is that tissue integrity is maintained and same tissue can be used for histopathological analysis.^40^, ^41^ Chae et al recently utilized HRMAS based metabolic profiling of breast tissue in distinguishing ductal carcinoma in situ lesions with and without invasive components.^87^ The GPC/PC ratio as well as the concentration of succinate and myo‐inositol was higher in the pure ductal carcinoma *in situ* group than in the ductal carcinoma *in situ* accompanying invasive carcinoma group. The orthogonal partial least square discriminant analysis models built with metabolic profiles clearly discriminated the two groups.^87^ Haukaas et al classified the metabolic profiles obtained using HRMAS MR spectroscopy in three different metabolic clusters and combined them with gene and protein expression data.^88^ Their results showed three metabolic clusters; cluster 1 having the highest levels of GPC and PC, cluster 2 was characterized with highest level of glucose, while cluster 3 showed the highest levels of alanine and lactate.^88^ Pathway analysis of metabolites and gene expression data indicated differences in glycerophospholipid glycolysis and gluconeogenesis metabolic pathways between the clusters. Genes related to extracellular matrix and collagens were down‐regulated in metabolic cluster 1 and unregulated in cluster 2 and 3, implying the differences in protein subtypes within the metabolic clusters.^88^ From India, recently, Paul et al. evaluated the differences in lipid profile of malignant breast tissues, lymph nodes and benign breast tissues.^89^ Their study reported reduced lipid content along with a higher fraction of free fatty acids in malignant breast tissues.^89^ Giskeødegård et al. investigated the relationship of metabolic profiles of breast cancer tissue with 5 year survival of breast cancer patient.^90^ The metabolic profiling of excised tissue was performed using high‐resolution MR spectroscopy. In a subgroup of ER positive patients, higher levels of lactate and glycine showed association with lower survival rates. These metabolites were suggested for predicting prognosis in breast cancer.^90^

## 4 IN VITRO ^1^H MRS

In vitro MRS studies of tissue extracts, axillary nodes and fine needle aspirates have also been reported to find out biomarkers for diagnosis and in evaluating the biochemistry of breast tumors. These studies have provided a further understanding of the metabolic differences between malignant and normal tissues by detecting simultaneously large number of metabolites that could not be observed in vivo.^42^, ^43^, ^44^, ^45^, ^46^, ^47^, ^48^, ^49^, ^50^, ^51^, ^52^ Further, the quantification of metabolites is comparatively easier using in vitro MRS than in vivo.^48^, ^49^

### 4.1 MRS of breast tissues, axillary nodes, and blood sera

The nuclear magnetic resonance (NMR) spectroscopic metabolic profiling of breast tissues obtained either by surgery or biopsy requires extraction of tissue metabolites. In general, the extraction of water soluble metabolites is carried out using perchloric acid extraction procedure.^91^, ^92^ Extraction of both water soluble and lipid soluble metabolites is performed using chloroform‐methanol‐water extraction procedure. The details of methodologies have been described elsewhere.^91^, ^92^ Sample collection and storage are important steps in metabolomics studies, requiring standardized protocols to be followed, since concentration of metabolites like lactate may change due to degradation of sample on exposure to room temperature during storage or sample processing.^93^, ^94^ Inappropriate handling of samples can result in high variability and inaccuracy in metabolite levels.^93^, ^94^

**FIGURE 8:**
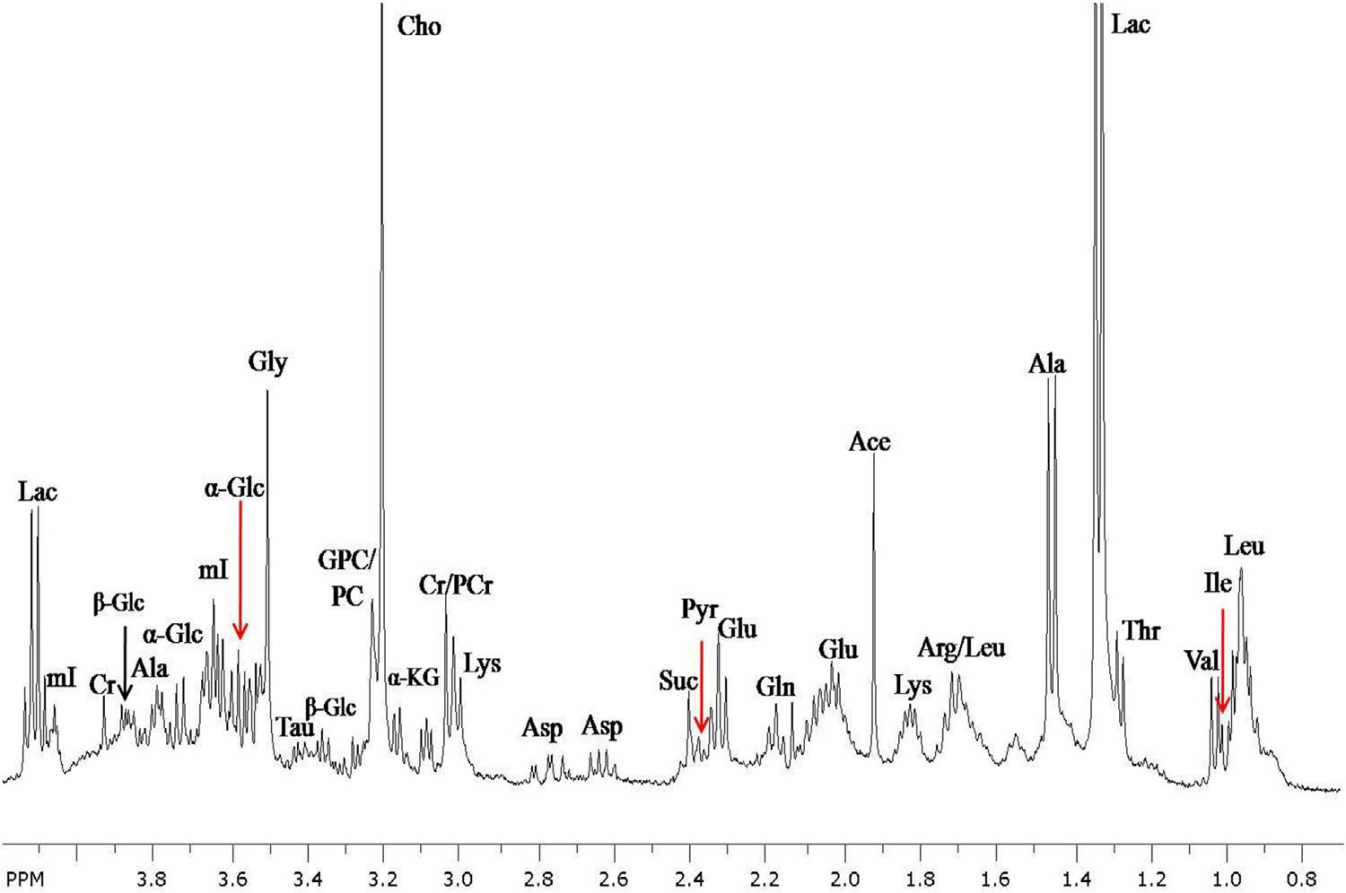
In vitro ^1^H magnetic resonance (MR) spectrum from the aliphatic region of the perchloric acid extracted from involved breast cancer tissue recorded at 400 MHz nuclear magnetic resonance (NMR). Abbreviations: Ala, alanine; Ace, acetate; Arg, arginine; Asp, aspartate; Cho, choline; Cr, creatine; Glc, glucose; Glu, glutamate; Gln, glutamine; GPC, glycerophosphocholine; Gly, glycine; Iso, isoleucine; KG, ketogultarate Lac, lactate; Leu, leucine; Lys, lysine; mI, myo‐inositol; PCr, phosphocreatine; PCho, phosphocholine; Pyr, pyruvate; Suc, succinate; Tau, taurine; Val, valine (Figure as originally published in reference # 52: Jagannathan NR, Sharma U. Breast tissue metabolism by magnetic resonance spectroscopy. *Metabolites* 2017;7(2):25. https://doi.org/10.3390/metabo7020025)

Gribbestad et al reported first in‐vitro high‐resolution proton MRS study of normal (non‐involved) and malignant (involved) breast tissues following perchloric acid extraction.^42^ The non‐involved tissues showed predominant signals from glucose and other carbohydrates, while these compounds were in low levels in involved tissues. Tumor extracts were characterized with high concentrations of taurine, lactate, succinate, and Cho and very low level of phosphocreatine. We also reported the metabolic profile of extracts of involved and non‐involved breast tissues using high‐resolution in vitro proton MRS (Figure 8).^43^, ^95^ Significantly higher concentration of several metabolites like lysine, alanine, glutamine, glutamic acid, phosphocreatine+creatine, Cho, GPC+PC, acetate lactate, and myo‐inositol was seen in involved tissues compared to the non‐involved tissues. These metabolites showed five‐ to tenfold increase in malignancy.^43^ No significant differences in the levels of phenylalanine, tyrosine, formate, and glucose between cancerous and non‐involved tissues was observed. Beckonert et al using in‐vitro ^1^H MRS of tissue extracts in combination with pattern recognition approach reported that the metabolic profile of tumors is related to tumor grade.^44^ They reported significant differences in uridine di‐phosphate‐hexose, phosphoethanolamine and PC between tumors with different grades. Malignant tumors had higher levels of taurine and lipid metabolites, while glucose and myoinositol were in higher level in controls.

These findings indicated changes in metabolism associated with breast tumor formation. It is known that in normal cells the main energy substrate is glucose that is utilized through glycolytic pathway followed by tricarboxylic acid cycle and oxidative phosphorylation for energy generation in the presence of oxygen.^96^ However, in cancer cells, the rate of glycolysis is increased and pyruvate, a product of glycolysis is converted to lactate, thus enhancing the level of this metabolite even in the presence of sufficient oxygen levels.^97^, ^98^ It has been reported that several intermediate compounds of glycolytic pathway are used as substrates for synthesis of other molecules through various biosynthetic pathways like pentose phosphate pathways. For example, ribose‐phosphate is an intermediate of glycolytic pathway is utilized for the synthesis of nucleic acids through pentose phosphate pathway. Since, tumor cells have rapid rate of cell division, there is requirement of larger pool of substrates for biosynthesis of other molecules like nucleic acid. Thus, this larger pool of substrate is achieved by increasing the rate of glycolysis in tumor cells by dysregulating several molecular pathways.^98^, ^99^, ^100^ The higher concentration of several amino acids like alanine, glutamine, lysine, and glutamate in tumor cells.^95^ Amino acids have been found to have various roles in cell metabolism. They can be utilized as energy substrate, as anti‐inflammatory agents and act as regulators as required for maintenance and cellular growth. It has been reported that glutamate and glutamine are utilized as an energy source by entering into TCA cycle in tumor cells.^101^, ^102^, ^103^ An important antioxidant glutathione is also synthesized using glutamate as substrate. Amino group of glutamate is also used in the synthesis non‐essential amino acids such as alanine, aspartate, glycine, and serine.^101^, ^102^

Axillary node metastasis is an important prognostic factor in breast cancer management. Generally, axillary node dissection is the standard part of surgery for breast cancer patients. However, this can be avoided if there are no micro‐metastases in nodes. Therefore, detection of micro‐metastases may have an important role in the prognosis of breast cancer. We investigated the metabolite composition of axillary lymph nodes using various one‐ and two‐dimensional in vitro NMR methods in patients with breast cancer.^48^ The comparison of metabolic profile of involved and non‐involved nodes showed significantly higher concentration of GPC+PC in involved nodes. This indicated the increased membrane synthesis in rapidly dividing cancer cells. The level of lactate was also higher in the involved nodes, indicating a higher rate of glycolysis in tumor cells.^104^ Our study further showed that nodes with metastases have increased ratio of metabolites [(GPC+PC)/threonine. The use of this as a marker of malignancy detected axillary node metastases with a sensitivity of 88% and a specificity of 91%.^49^ A higher lactate level has also been suggested as an indicator of the presence of malignant cells in a study that used MRS of lymph nodes excised from nude mice.^103^ Mountford et al reported the MRS study of nodes from tumor bearing rats for detecting micro‐metastases and reported that MRS detected micro‐metastases that were missed by conventional histopathology.^103^

A study by Singh et al reported the metabolomics of serum of breast cancer patients.^50^ They reported that patients with high expression of inositol 1,4,5 trisphosphate receptor were having higher levels of alanine, lactate, lysine, and lipoprotein content compared to healthy subjects. The levels of glucose and pyruvate were lower in sera of breast cancer patients compared to healthy subjects.^50^ Few studies have also reported differences in the metabolic profile of serum of breast cancer patients in early stage and metastatic breast cancer.^51^, ^105^ Significant differences were seen in metabolites such as glutamate, lysine, beta‐hydroxybutyrate, glucose, lactate, and N‐acetyl glycoprotein in early and late stage cancer indicating the role of these metabolites in cancer progression.^51^

Richad et al investigated the metabolome of blood plasma of 50 early breast cancers and 15 metastatic breast cancer patients by ^1^H‐ NMR spectroscopy.^106^ Using multivariate analysis, levels of several plasma metabolites including glucose, pyruvate, lactate, alanine, acetate, β‐hydroxy‐butyrate,acetoacetate, isoleucine, leucine, glutamate, glutamine, lysine, valine, threonine, glycine, phenylalanine, tyrosine, urea, creatine, and creatinine were found to be modulated between patients with early and metastatic breast cancer.^106^ Additionally, lactate levels were inversely correlated with the tumor size. It was suggested that metabolism is altered even at early stages of breast cancer.^106^Flote et al characterized serum lipid profiles of breast cancer patients using NMR spectroscopy.^107^ They documented inverse associations between HDL phospholipids and Ki67, specifically in between HDL1's contents of phospholipids, cholesterol, apolipoproteins‐A1, A2, and Ki67.^107^

### 4.2 MRS of fine needle aspirates

**FIGURE 9:**
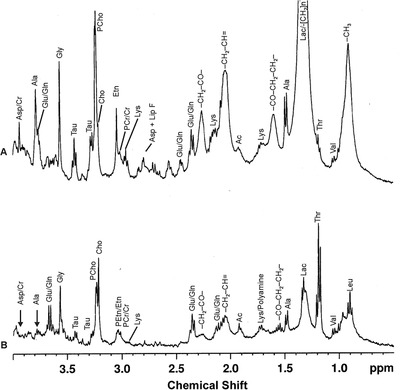
High‐resolution 400 MHz proton MR spectrum of fine‐needle aspirate (FNAC‐ex vivo) from (**A**) malignant and (**B**) benign breast tissues. Abbreviations: Leu: leucine; Val: valine; Thr: threonine; Lac: lactate; Ala: alanine; Lys: lysine; Ac: acetate; Glu: glutamic acid; Gln: glutamine; PCr: phosphocreatine; Cr: creatine; PEtn: phosphoethanol amine; Etn: ethanol amine; PCho: phosphocholine; Cho: choline; Tau: taurine; Gly: glycine; Asp: aspartate; CH_3_, CH_2_CO, CO‐CH_2_‐CH_2_‐: groups of lipids (Reprinted with permission from Bentham Science Publishers from reference # 43)

The fluid obtained through aspirations of lesions has also been examined in few studies and by our group to understand its utility in identifying biomarker/s of breast cancer.^43^, ^45^, ^46^, ^47^ Usually, two aspirations from a lesion are taken after palpation of the tumor to obtain appropriate quantity of the sample for in‐vitro high‐resolution NMR studies of FNAC samples. In a study from our laboratory the metabolic profile of FNAC sample of patients with IDC showed increased levels of many metabolites in addition to choline compared to benign aspirates (Figure 9).^43^ This is in agreement with the observation of elevated tCho levels seen using in‐vivo MRS in breast cancer patients. Significant differences in the metabolic profile were also seen in other breast cytopathologies.^43^ Use of MRS of FNAC samples has been reported in discriminating invasive cancer, normal, and benign tissues on the basis of tCho levels.^46^ Mackinnon et al performed ^1^H MRS of FNAC samples from benign and malignant lesions and documented that choline to creatine ratio (Cho/Cr) has a 95% sensitivity and a 96% specificity for discriminating malignant from benign lesions.^45^ Similarly, a three‐stage statistical classification strategy analysis has been reported for diagnosis and prognosis of breast cancer.^47^ It distinguished malignancy with a 93% accuracy from the benign disease.^47^

### 4.3 MRS of cell lines

The metabolomics studies of breast cancer cell lines have also been found as a valuable platform for discovery of biomarkers and understanding the molecular mechanism of cancer progression and response to therapy.^8^, ^9^ Mori et al have studied the choline and lipid metabolism in breast cancer cell lines.^108^ An increase of PC and tCho as well as alterations in lipids have been consistently shown in cancer cells and tissue. They have compared the metabolic profile and protein expression of enzymes regulating Cho and lipid metabolism in breast cancer and prostate cancer cell lines.^108^ Significant differences in lipid and Cho metabolism and expression of proteins were reported in both the breast and prostate cancer cells. In another study, authors used targeted silencing of two glycerophosphodiesterase genes, GDPD5 and GDPD6 by small interfering RNA (siRNA) for studying choline and phospholipid metabolism in MDA‐MB‐23 and MCF‐7 cell lines of breast cancer.^109^ It was reported that silencing of GDPD6 increased the GPC levels more than GDPD5 silencing, suggesting this as a potential treatment strategy for breast cancer.^109^ Gowda et al using NMR metabolomics approach studied the effect of inhibition of glutaminase by using the inhibitor BPTES (bis‐2‐(5‐phenylacetamido‐1,3,4‐thiadiazol‐2‐yl)ethyl sulfide) in two breast cancer cell lines MCF7 and MDA‐MB231, cancer proliferation is found to be associated with glutamine addiction.^108^ The inhibition of glutaminase was found to affect several metabolic pathways including glycolysis, Kreb's cycle, amino acid and nucleotide metabolism, the metabolic changes were more pronounced in MCF7 cells with alterations in 14 metabolites compared to seven for MDA‐MB231.^110^ Armiñán et al investigated the changes in metabolomic profiles in response to doxorubicin and N‐(2‐hydroxypropyl) methacrylamide (HPMA) copolymer‐conjugated form in an in‐vitro cell culture model and also in an in vivo orthotopic breast cancer model along with protein expression.^111^ They found that polymer conjugation led to increased apoptosis, reduced phospholipids and glycolysis compared to the free doxorubicin.^111^ Bhute et al investigated the effect of veliparb and radiation on three breast cancer cell lines and reported that therapy induced metabolic changes are cell line dependent.^112^ Nitrogen metabolism, aminoacyl‐tRNA biosynthesis, taurine and hypotaurine metabolism, glycine, serine, and threonine metabolism were found to be enriched by pathway enrichment and topology analysis after therapy in all three breast cancer cell lines. These metabolic changes were cell line‐dependent, indicating the importance of different metabolic responses in different cancer sub‐types.^112^

## 5 SUMMARY, OUTLOOK, AND FUTURE DIRECTIONS

MR spectroscopy and MS play an important and complementary role by measuring large number of metabolites in various biological matrices, providing valuable information for diagnostic applications, and for translational research. Compared to MS, the high reproducibility, minimal sample preparation, non‐selective and non‐destructive nature, and the ability to identify the unknown metabolites have made MRS an important tool for various metabolomics applications. This review briefly discussed the use of MRS based metabolomics approach in identifying biomarkers for diagnosis and therapeutic outcome in breast cancer, especially focusing on the various works carried out in India. The usefulness of ex vivo HRMAS MRS of intact tissue, in vitro MRS of tissue extracts, axillary nodes, and FNAC samples in the biochemical characterization of breast tissues was presented. These studies have shown that various metabolic pathways, including glycolysis, amino acid metabolism, and membrane metabolism have been altered in the breast cancer. The potential of metabolomics approach using in vitro NMR studies of various biological matrices has provided wide coverage of metabolites. Further, these studies have provided a more comprehensive understanding of the metabolic profile of the tumor tissues. However, there are only limited numbers of studies in the literature. There is a need to perform studies in a large cohort of patients, including various histological and molecular subtypes of malignant and benign breast lesions. This will provide a better understanding of the mechanisms of malignant transformation, identifying new therapeutic targets, better biomarkers for early diagnosis, and response assessment. Additionally, various in vivo MRS studies have documented altered lipid composition, water‐fat ratio, fat fraction, and tCho levels determined using in vivo MRS as promising biomarkers for diagnosis and for monitoring early response to therapy. Early diagnosis of the disease and the prediction of early response of tumor is important in better management of the disease, enabling surgical options, and open the possibility of alternative therapy for non‐responding patients. Studies have investigated the molecular mechanism of tCho elevation and its association with the molecular heterogeneity of breast lesions. There is need to perform large scale multi‐center studies, which would also help in comparing the data of different centers and discovery of robust biomarkers. Moreover, analysis of various biological matrices from breast cancer patients using high‐throughput analytical platforms MS and MRS have identified a large number of metabolites as potential candidates to serve as biomarkers, however, these state‐of‐the‐art methodologies are still confined to research laboratories. There is a significant scope to offer simplistic and reproducible markers that may be used in complementing existing diagnostic techniques in clinics for providing personalized health care. Nevertheless, there is a need to perform systematically designed metabolomics studies of biological matrices like blood sera/plasma and urine to discover robust non‐invasive biomarkers for concerning issues of early diagnosis, stratifying high‐risk patients, monitoring therapy, understanding drug‐resistance, recognizing new therapeutic targets and developing better therapeutic approaches.

## CONFLICT OF INTEREST

The author declares no conflict of interest.

## Data Availability

Data sharing is not applicable to this article as no new data were created or analyzed in this study.
